# Shapeshifter W-Tau Peptide Inhibits Tau Aggregation and Disintegrates Paired Helical Filaments

**DOI:** 10.1021/acs.biochem.4c00809

**Published:** 2025-03-27

**Authors:** Indalo Domene-Serrano, Raquel Cuadros, Vega García-Escudero, Francisco Vallejo-Bedia, Ismael Santa-María, Laura Vallés-Saiz, Félix Hernandez, Jesús Avila

**Affiliations:** † Centro de Biología Molecular Severo Ochoa, CSIC-UAM, Madrid 28049, Spain; ‡ Facultad de Ciencias Experimentales, Universidad Francisco de Vitoria, Pozuelo de Alarcon, Madrid 28223, Spain; § Center for Networked Biomedical Research on Neurodegenerative Diseases (CIBERNED), Instituto de Salud Carlos III, Madrid 28029, Spain; ∥ Departamento de Anatomía, Histología y Neurociencia, School of Medicine, Autonoma de Madrid University (UAM), Arzobispo Morcillo, 4, Madrid 28029, Spain

## Abstract

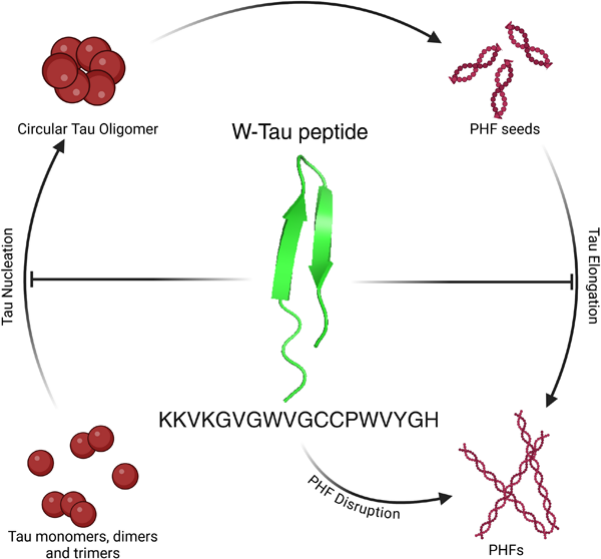

Tauopathies comprise a range of neurodegenerative conditions characterized by the aberrant accumulation of tau protein clumps in the brain. These aggregates are formed by different tau splicing isoforms. Here, we analyzed the role of a specific intron-derived peptide called the W-Tau peptide on the polymerization–depolymerization of tau filaments. This peptide originates from a new isoform of the tau protein, named W-Tau, which is formed due to the retention of intron 12. AlphaFold3 (AF3)-based *in silico* investigations suggested that the W-Tau peptide interacts with tau monomers. Our *in vitro* experiments confirmed these predictions and showed that the W-Tau peptide inhibited tau aggregation. In addition, the W-Tau peptide disrupted preexisting paired helical filaments (PHFs) isolated from postmortem brain samples of patients with Alzheimer’s disease, thereby supporting its potential therapeutic value. The effectiveness of the W-Tau peptide was demonstrated by the decrease in tau aggregation observed after cotransfection of the W-Tau peptide and PHF seeds, as demonstrated by analysis involving a fluorescence resonance energy transfer (FRET) cell biosensor. The W-Tau peptide breaks PHFs by selectively attaching to their ends, causing the structures to unwind and convert into circle-like formations. Considering the potential neuroprotective effects against tauopathies, the W-Tau isoform and its peptide are interesting candidates for future therapeutic interventions.

## 1 Introduction

Alzheimer’s disease (AD) is the most common form of dementia among elderly individuals worldwide.^1,2^ Several behavioral abnormalities, including memory loss, cognitive decline, sleep disorders, and neuropsychiatric problems, are indicative of AD.^3−5^ From a molecular perspective, the histopathological hallmarks of AD include the formation of intracellular neurofibrillary tangles (NFTs)^6−9^ made up of abnormal depositions of misfolded microtubule-associated protein-tau isoforms and extracellular senile plaques made up of misfolded and aberrantly accumulated amyloid-beta (Aβ) peptides^10−14^

Focusing on AD, as a tauopathy, it is important to know that tau protein (UniProtKB P10636) is encoded by the MAPT gene located on chromosome 17q21.31^15^ and is comprised of 16 exons.^16,17^ Alternative splicing of nuclear MAPT RNA yields several tau isoforms having different sizes and functions.^18−23^ Exons 4A and 6 are mainly found in the mRNA of the peripheral nervous system (PNS).^24,25^ In the central nervous system (CNS), alternative splicing of exons 2, 3, and 10 results in six different tau isoforms that range from 352 to 441 residues in length.^18,26−28^ The six main CNS tau isoforms (0N4R, 1N4R, 2N4R, 0N3R, 1N3R, 2N3R) contain 0–1–2 N-terminal inserts and either 3 or 4 microtubule-binding repeats (MTBRs), with the 4R variants having higher microtubule-binding affinity than the 3R variants.^17,29,30^ In the fetal human brain, only the 0N3R isoform is expressed, whereas the adult brain expresses all six isoforms.^31^ In the human adult brain, 4R- and 3R-tau are generally present in equimolar quantities^17,30^ and divergence from this ratio in aberrant tau polymers may be characteristic of tauopathies such as neurodegenerative frontotemporal dementia, Pick’s disease, or corticobasal degeneration.^17,32^ In addition, structural differences were found in tau polymers present in some different tauopathies with 4R and 3R tau.^33^

Tau isoforms, arising from differences in the presence of specific exons, lead to conformational changes in the three-dimensional structure of the protein. These changes may result in an increase in the distance between the terminals of the protein and in more globular or more fibrillar structures.^34^ Previously, tau has been described as an intrinsically disordered protein (IDP),^35^ and the structural changes introduced during alternative splicing have consequences on the functional versatility of tau.^32^ Tau protein may acquire local structures such as α-helices, β-sheets, and polyproline-II helices in various sequence segments.^36−38^ Additionally, a “paperclip’’ model for tau 3D structure has been suggested based on a fluorescence resonance energy transfer study^39^ wherein the N-terminal, C-terminal, and repeat domains are folded in such a manner that these regions approach each other.^39,40^ Unlike a well-folded protein whose structure restricts binding to only one type of ligand, tau is likely to adopt multiple conformations in a context-dependent manner.^41^

Recently, our laboratory described a new tau isoform generated by the retention of intron 12 of the human MAPT gene.^42^ Shortly after the initiation of intron 12 of the human MAPT gene, a stop codon appears, followed by a canonical polyadenylation sequence, resulting in truncation of the protein at this point. Thus, the W-Tau isoform differs from other human tau isoforms in that it lacks exon 13 of the MAPT gene and includes an 18-amino-acid sequence corresponding to the translation of the retained fragment of intron 12 in its place, at its carboxyl-terminal region, immediately after exon 12. The 18-residue sequence contains two tryptophan residues (W), an amino acid that cannot be found at any other location in the human tau sequence. Retention of the beginning of intron 12 and the truncation of exon 13 result in neuroprotective properties of W-Tau isoforms, such as a lower aggregation capacity or the ability to inhibit the polymerization of other tau isoforms.^42^ Additionally, data from our laboratory have shown that these 18 amino acids (W-Tau peptide) exhibit properties similar to those of the complete isoform,^43^ but the mechanism is not yet known. This peptide is also present in another new tau isoform, generated by the retention of both introns 3 and 12.^44^

In this study, we further investigated the role of the W-Tau peptide in decreasing tau aggregation. We evaluated both *in silico*, using the structures proposed by the AlphaFold3 (AF3) machine learning protein prediction program, and *in vitro* through biochemical assays and in cellular cultures. *In vitro* molecular experiments were performed by simulating the possible chronological order of events leading to tauopathy, starting with the aggregation of soluble tau monomers, progressing through seeding activity, and ultimately leading to the formation of paired helical filaments (PHFs) that form larger structures known as neurofibrillary tangles (NFTs).^45,46^ Thus, we studied the effect of the W-Tau peptide in an *in vitro* aggregation assay.^47^ Our analyses indicated that the W-Tau peptide not only prevents tau aggregation but also facilitates the disintegration of human tau polymers.

## 2 Material and Methods

### 2.1 Protein, Peptides, and W-Tau Antibody

Experimental studies were conducted in Microtubule-associated protein Tau Human, UniProtKB: P10636. The W-Tau peptide (KKVKGVGWVGCCPWVYGH)—KK from exon 12 and VKGVGWVGCCPWVYGH from intron 12—was obtained from Abyntek Biopharma S.L. (Parque Tecnológico de Bizkaia, Derio, Spain). The Control peptide (KYVPGVVGCFGKHVGCFK) was obtained from NZYTech, Lda (Estrada do Paço do Lumiar—Campus do Lumiar, Lisboa, Portugal). The compared sequences of both peptides are indicated in Figure S1. All the peptides were dissolved in sterile H_2_O milli-Q. The antibody against the W-Tau peptide, named W-Tau antibody, was obtained from Abyntek Biopharma S.L. (Parque Tecnológico de Bizkaia, Derio, Spain).

### 2.2 3D Modeling Analysis

AlphaFold3 server^48^ was used for the structure prediction of the Tau 4R2N microtubule-binding domain with the W-Tau peptide. Visualization of protein structures was performed with The PyMOL Molecular Graphics System, Version 2.0, Schrödinger, LLC.

### 2.3 Tau 4R2N Purification

Tau 4R2N was purified following previously established methods.^49^

### 2.4 Fibril Formation

Tau 4R2N 20 μM was incubated in the absence or presence of 5 μM W-Tau peptide or 5 μM Control peptide.^47^ After incubation, samples were visualized by electron microscopy.

### 2.5 Electron Microscopy

The drop vapor diffusion experiments were visualized under a JEM1400 Flash Transmission Electron Microscope (JEOL) using 400 MEs Copper Collodion grids ionized in a BAE 120 Evaporator (Bal-Tec). For the passage of the samples to the grids, they were adsorbed for 5 min, cleaned with H_2_O milli-Q and left for 40 s in 2% uranyl acetate. Data of tau filaments and oligomers was analyzed using the image processing and analysis software ImageJ (ImageJ, NIH).

### 2.6 Human Brain Samples

Brain samples for PHF purification from sporadic Alzheimer’s disease patients were kindly provided by Dr. A. Rabano from Banco de Tejidos (Fundación CIEN, Instituto de Salud Carlos III, Madrid, Spain). Based on quantitative pathological features, the Alzheimer’s brain specimens were classified according to Braak stages. Written informed consent was obtained premortem from all patients.

### 2.7 PHF Seeds’ Purification

The method of Dujardin was used.^50^ Homogenization of 300 mg of human brain tissue was performed in 1.5 mL of phosphate-buffered saline (PBS) with complete, EDTA-free protease inhibitor cocktail tablets (11873580001, Roche Diagnostics Deutschland GmbH). The tissue was added to the pestle, followed by the addition of PBS. Homogenization was carried out using a pestle, applying strong pressure in a spiral motion. After homogenization, the mixture was transferred to an Eppendorf tube and centrifuged for 10 min at 10,000 rpm at 4 °C using a rotor F-45-12-11. The supernatant was carefully collected without disturbing the pellet. The protein concentration of the supernatant was determined by BSA analysis. Once measured, the percentage of tau protein in the sample was calculated based on the fact that tau protein constitutes 0.1–0.5% of the total protein in the brain^51^ The supernatant was subjected to ultracentrifugation for 30 min at 150,000 *g* at 4 °C using a rotor TL100.3. The proportion was calculated to ensure that each aliquot of the extract contained 800 ng of tau. The resulting pellet was collected and resuspended in 50 μL of PBS. The suspension was stored at −80 °C for further use. It should be taken into account that there is an equilibrium among tau polymers, oligomers, and monomers, and that the presence of heparin displaces that equilibrium to polymer formation.^52^

### 2.8 FRET Aggregates

Tau RD P301S FRET Biosensor (ATCC CRL3275),^53^ known as the FRET cell line, was cultured in DMEM supplemented with 10% fetal bovine serum, 2 mM glutamine, nonessential amino acids, 10 U/mL penicillin, and 10 μg/mL streptomycin (supplemented DMEM), at 37 °C and 5% CO_2_. The FRET cell line was seeded in M96 at 60% confluence. To produce tau aggregates, these cells were transfected with PHFs seeded from Alzheimer’s patients’ brains. Transfection was performed by mixing 0.5 μL of Lipofectamine 2000 (Thermo Fisher, 11668027) plus 100 μL of OptiMem on one side and 0.5 μL of seeds together with 100 μL of OptiMem on the other side. Each tube was left to incubate for 5 min for further mixing and then incubated for 20 min. The transfection medium was added to the cells. Along with this transfection, incubations with the W-Tau peptide were performed.

### 2.9 Optical Microscopy

Visualization of FRET cell line PHF seed aggregates was performed in our *in vivo* imaging system (Axiovert 200 inverted microscope (Zeiss) coupled to a monochrome sCMOS camera) at a constant temperature of 37 °C and 5% CO_2_. The extent of the tau-positive FRET signal was analyzed using the image processing and analysis software ImageJ (ImageJ, NIH).

### 2.10 *In Vitro* Polymer Aggregation Determination

Drop vapor diffusion was used to analyze the inhibitory capacity of W-Tau peptide on tau polymer aggregation *in vitro*. PHF seeds (1 μL), in the absence or presence of 5 μM heparin (average relative molecular weight is 15 kDa) as a polymerization inducer, were incubated in Buffer A (0.1 M MES (pH 6.4), 0.5 mM MgCl_2_, and 2 mM EGTA). To this mixture, 5 μM W-Tau peptide or the Control peptide was added. After 72 h of incubation at room temperature, samples were visualized by transmission electron microscopy.^54^

### 2.11 PHFs’ Purification

PHFs were purified following a previously established method by Greenberg.^55^ Brain samples stored at −70 °C were retrieved and transferred onto dry ice. Approximately 10–20 g of brain tissue was weighed for further processing. The tissue was homogenized in 10 volumes of Buffer H (10 mM Tris, pH 7.4, 1 mM EDTA, 0.8 M NaCl, and 10% sucrose). Centrifugation was carried out at 13,000 *g* for 20 min at 4 °C using a GSA Centrifuge Fixed-Angle Rotor 6 × 250 mL, and the supernatant (SN) was collected. The solution was adjusted to a 1% concentration of *N*-lauroylsarcosine sodium and 0.1% DTT at room temperature (RT), with adjustments made according to the volume of the supernatant. Incubation with agitation was performed at 37 °C for 2–2.5 h. Centrifugation was then conducted at 35,000 rpm for 35 min using a Sorvall A641 rotor. The reaction was stopped and stored at 4 °C, or the experiment proceeded to the next step. Homogenization of the pellet was performed using 5–10 mL of Buffer H containing 1% CHAPS and 1% DTT. The homogenate was centrifuged at 35,000 rpm for 1 h using a Sorvall A641 rotor. The resulting pellet was resuspended in 3–4 mL of Buffer H with 0.1% DTT. A discontinuous sucrose gradient in Buffer A (0.1 M MES (pH 6.4), 0.5 mM MgCl_2_, and 2 mM EGTA) was prepared, consisting of 4 mL of 50% sucrose and 3.5 mL of 35% sucrose. Centrifugation was performed at 35,000 rpm for 2 h using a Beckman Ti 41 rotor. The interface before the 50% layer was collected. Concentration was achieved by centrifugation at 100,000 *g* for 1 h at 25 °C using a TL100.3 rotor. The resulting pellet was resuspended in 400 μL of 10 mM phosphate buffer with 0.1 M NaCl. Aliquots were prepared and stored at −20 °C.

### 2.12 *In Vitro* Incubation of Human Derived PHFs with W-Tau Peptide

Purified PHFs (1 μL) were incubated with 5 μM W-Tau peptide using the technique of Vapor Diffusion Drop in Buffer A. The drops were left for 24 h at room temperature. After incubation, the polymers were analyzed under a transmission electron microscope and analyzed by a turbidity assay at 350 nm.

### 2.13 Immunogold Labeling

Purified PHFs (1 μL) were mixed with 0.5 μM W-Tau peptide. Without incubation time, drops were adsorbed for 5 min onto 400 MEs Copper Collodion grids ionized in a BAE 120 Evaporator (Bal-Tec) and washed with PBS for 3 min. Grids were blocked for 10 min with PBS + 10% FBS and then incubated with W-Tau antibody diluted in PBS + 5% FBS for 30 min at RT. They were washed once in PBS for 3 min, then incubated with 5 nm colloidal gold conjugated with a secondary antibody diluted 1:30 in PBS and 5% fetal bovine serum (FBS) for 30 min. The grids were washed once in PBS for 3 min and once in H_2_O milli-Q for 3 min, then dried and stained with uranyl acetate for 40 s. The samples were visualized by transmission electron microscopy.

### 2.14 Data Analysis

For quantitative experiments, the statistical significance was determined by Student’s *t* test using GraphPad Prism (GraphPad Software, La Jolla, CA, USA). One-way ANOVA with Tukey post hoc test was applied for comparisons involving more than two groups. A *p*-value of less than 0.05 was considered significant. For all figures in which error bars are shown, data represent the mean ± SEM. Statistical outliers and specimens with measurement errors were excluded.

## 3 Results

### 3.1 Interaction between Tau Monomers and W-Tau Peptide

As previously demonstrated, the W-Tau isoform exhibits neuroprotective properties, including the inhibition of tau protein polymerization.^42,43^

**Figure 1:**
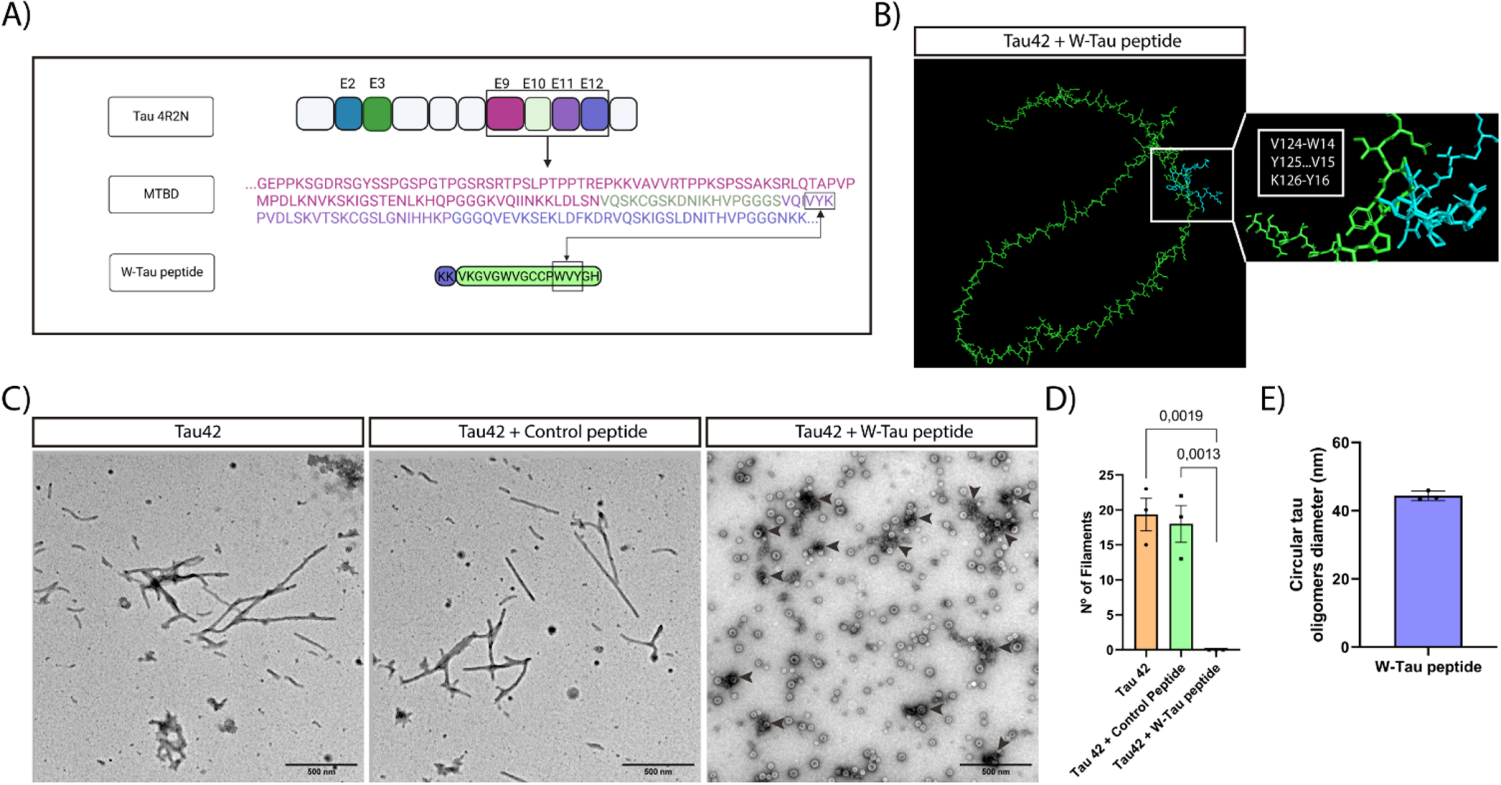
AF3 prediction of interaction between the W-Tau peptide and Tau microtubule-binding domain and transmission electron microscopy of Tau 4R2N in the presence or absence of the W-Tau peptide. (A) Scheme showing isoform Tau 4R2N, to microtubule-binding domain (MTBD) and W-Tau peptide sequences. Scheme created with BioRender. (B) Three-dimensional structure of the MTBD (green) and W-Tau peptide (blue) interaction by AF3. (C) Representative electron microscopy images of Tau 4R2N filamentous structures and more circular alike structures of Tau 4R2N with the W-Tau peptide. Black arrows point out each Tau circular oligomer. (D) Number of Tau 4R2N filaments found per field. Quantitative analysis shows the mean ± SEM. *p*-vValue, represented in each graph, using two-way ANOVA followed by Student’s *t* test for comparisons. Scale bar of 500 nm. Sample size: *n* = 20 fields per replicate. Each single value in graphs represents each replicate from independent samples (technical replicates: *n* = 3). (E) Quantification of the diameter of circular oligomer structures found in the presence of the W-Tau peptide. Each single value in graphs represents each technical replicate.

The inhibitory capacity of the W-Tau peptide polymerization was first studied *in silico* to investigate the interactions between the peptide and the regions of tau aggregates (Figure 1B). First, the interactions between the W-Tau peptide and the microtubule-binding domain of 4R tau were analyzed using the AF3 protein structural predictor^48^ (Figure 1A). In this analysis, a possible interaction was found between the -VYK- amino acids of exon 11 from the tau monomer and the -WVY- amino acids of the W-Tau peptide (Figure 1B). The incubation of Tau 2N4R for 24 h at 37 °C with agitation led to the formation of filamentous structures. This incubation was carried out in the presence or absence of the W-Tau peptide and the Control peptide (Figure 1C,D), where the filamentous structures of Tau 2N4R were disrupted in the presence of the peptide, resulting in a circular conformation composed of tau protein oligomeric structures with an average diameter of 40–45 nm (Figure 1E).^56^

### 3.2 Tau Aggregation Inhibitory Effect of the W-Tau Peptide in FRET Biosensor Analysis

Upon observing the results obtained using the W-Tau peptide in molecular assays against tau *in vitro*, we proceeded to the analysis of the inhibition of tau protein aggregation in cell culture.

The cell culture chosen to analyze the inhibitory capacity of the W-Tau peptide was the Tau RD P301S FRET Biosensor cell line,^53^ hereafter referred to as the FRET cell line. Specifically, this cell line was generated by transducing two separate lentiviral constructs encoding Tau RD P301S-CFP and Tau RD P301S-YFP into human embryonic kidney 293T cells. The expression of these modified versions of tau remained soluble and monomeric, producing a negative FRET signal. By transfecting tau seeds (PHF seeds), intracellular tau protein aggregates are formed emitting a FRET signal that allows for quantification of the signal via microscopy or flow cytometry.^53^

**Figure 2:**
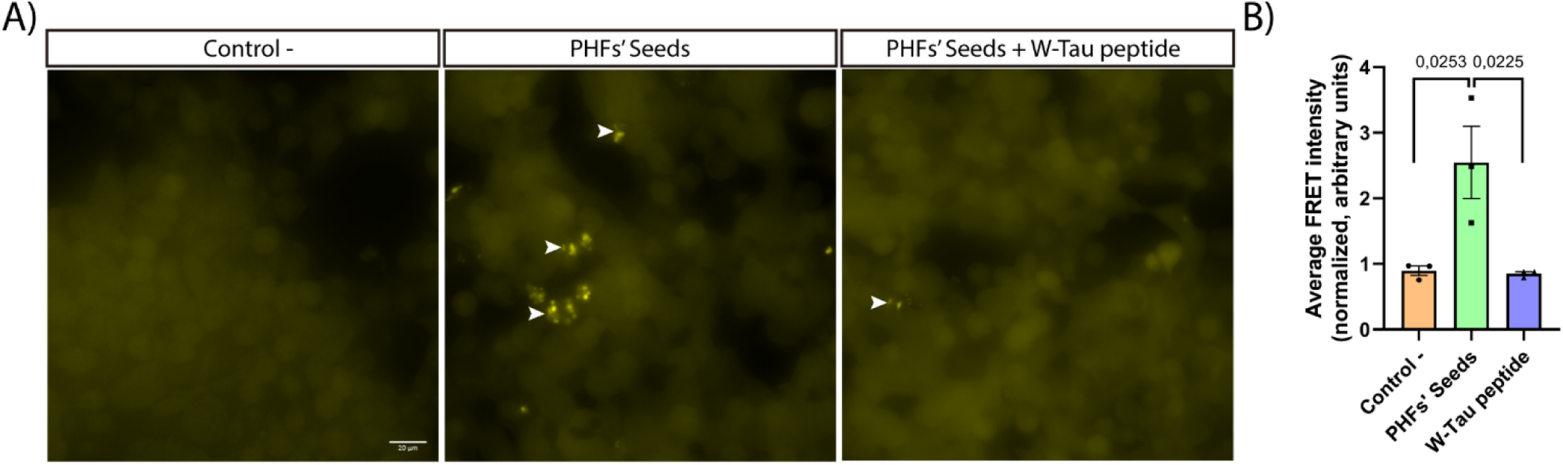
The W-Tau peptide inhibits Ttau aggregation in the FRET cell line. (A) Representative images at 48 h post-transfection showing the state of the aggregates in Control– (nontransfected), Control+ (transfected with PHF seeds), and W-Tau peptide (cotransfected with PHF seeds and the W-Tau peptide). White arrow heads point out tau aggregates. Scale bar of 20 μm. (B) Quantification of the aggregation state by measuring the fluorescence intensity of the FRET emission. Quantitative analysis shows the mean ± SEM. *p*-vValue, represented in each graph, using two-way ANOVA followed by Student’s *t* test for comparisons. Sample size: *n* = 10 fields per replicate. Each single value in graphs represents each replicate from independent samples (technical replicates: *n* = 3).

Using the aforementioned FRET cell line, we tested the efficacy of the W-Tau peptide. At 48 h post-transfection with PHF seeds, the highest peak intensity of the aggregates was observed, so the most representative images of the experiment were taken at this time (Figure 2A). In cells transfected only with the PHF seeds, an increase in the number and size of aggregates was observed. Cells cotransfected with the W-Tau peptide displayed a significant reduction in aggregate formation, as observed in Figure 2B, indicating the inhibitory effect of the W-Tau peptide.

### 3.3 W-Tau Peptide Inhibits Tau Filament Assembly and Elongation

**Figure 3:**
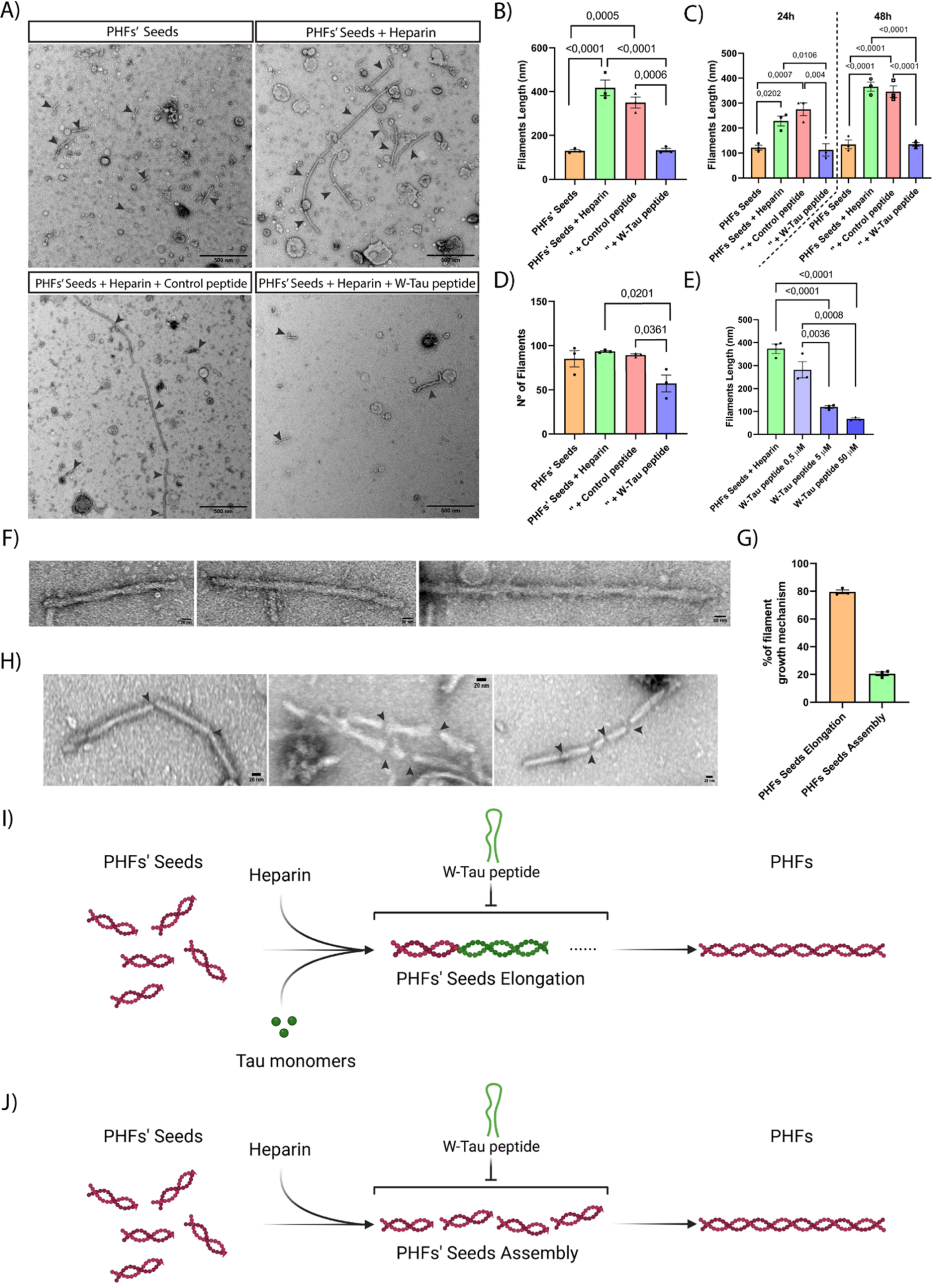
Inhibition of PHF seeds’ polymerization by the W-Tau peptide *in vitro*. (A) Representative images of the different analyzed samples: PHF seeds without heparin, PHF seeds with heparin, and PHF seeds with heparin and the W-Tau peptide or the Control peptide. Black arrow heads point out each PHF seed. (B) PHF seed filament length quantification. Quantitative analysis shows the mean ± SEM. *p*-vValue, represented in each graph, using two-way ANOVA followed by Student’s *t* test for comparisons. Sample size: *n* = 20 fields per replicate. Each single value in graphs represents each replicate from independent samples (technical replicates: *n* = 3). (C) Time-dependent (24 and 48 h) quantification of PHF seed filament length. (D) Number of PHF seed filaments per replicate. (E) Dose-dependent W-Tau peptide activity on filaments length. (F) Electron microscopy images of PHF seed elongation in the presence of heparin. This tau elongation is inhibited by W-Tau peptide. Scale bar of 20 nm. (G) Percentage of PHF seed growth mechanism. (H) Electron microscopy images of PHF seeds’ assembly in the presence of heparin. Black arrow heads point out each seed to seed link. This tau assembly is inhibited by the W-Tau peptide. Scale bar of 20 nm. (I) Illustration depicting PHF seed elongation in the presence of heparin and how the W-Tau peptide inhibits this process. (J) Diagram illustrating the assembly of PHF seeds in the presence of heparin and how the W-Tau peptide inhibits this process.

To determine whether the W-Tau peptide could block tau assembly, preexisting tau filaments obtained from the human brain (PHF seeds) were incubated in the presence of a tau protein polymerizing agent, such as heparin, and in the presence or absence of the W-Tau peptide. The results from Figure 3A demonstrated that the presence of heparin increased the length of the filaments formed by these PHF seeds. Conversely, the presence of the W-Tau peptide inhibited the elongation of these filaments, not only reverting them to the same length as that formed in the case of PHF seeds without the polymerizing agent but also slightly decreasing that length. The elongation was measured by analyzing the length of each filament (Figure 3B). This elongation process has time-dependent (Figure 3C) and dose-dependent (Figure 3E) response. Furthermore, in addition to inhibiting the polymerization of PHF seeds, a significant reduction in the number of filaments was observed in the presence of the W-Tau peptide (Figure 3D). This reduction suggests that the W-Tau peptide may depolymerize PHF seeds. Curiously, we saw how the assembly of PHF seeds could take place in opposition to normal tau elongation through tau monomers (Figure 3F). These small (seeds) filaments, in this case, in the presence of heparin, line up until they join together to form a longer filament, a paired helical filament (Figure 3H). Although we cannot exclude that fragmentation of preexisting filaments could occur, the proportion of both mechanisms is represented in Figure 3G. Diagrams illustrate the PHF seeds’ elongation mechanism (Figure 3I) and the assembly of PHF seeds (Figure 3J) in the presence of heparin and how the W-Tau peptide inhibits this process.

### 3.4 The W-Tau Peptide Can Disintegrate PHFs

After confirming the W-Tau peptide’s inhibitory activity on tau filament elongation and aggregation, we aimed to determine whether the W-Tau peptide could promote the depolymerization of PHFs.

**Figure 4:**
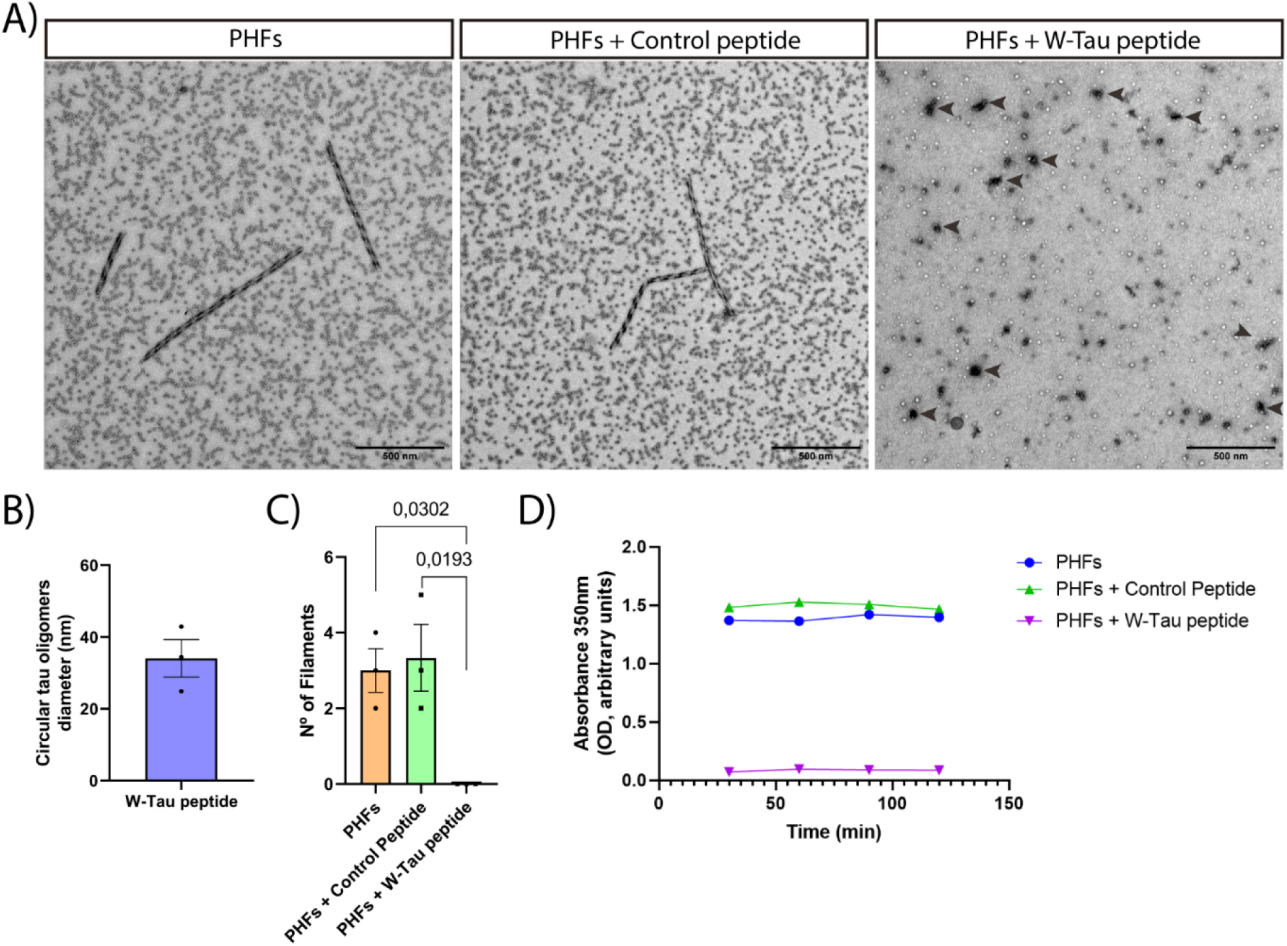
PHFs’ disruption by W-Tau Ppeptide. (A) Electron microscopy representative images of PHFs and the action of the W-Tau peptide and the Control peptide over PHFs at 24 h. Black arrows point out each circular tau oligomer. Scale bar of 500 nm. Sample size: *n* = 20 fields per replicate. Black dots correspond to ferritin shells that are present in PHF samples.^57^ (B) Quantitative analysis of circular tau oligomers found in the presence of W-Tau peptide at 24 h. (C) Number of paired helical filaments found per field. Each single value in graphs represents each technical replicate. Quantitative analysis shows the mean ± SEM. *p*-vValue, represented in each graph, using two-way ANOVA followed by Student’s *t* test for comparisons. Sample size: *n* = 20 fields per replicate. Each single value in graphs represents each replicate from independent samples (technical replicates: *n* = 3). (D) Absorbance analysis (350 nm) of conformational structure of PHFs in the absence or presence of W-Tau peptide at different concentrations.

For this purpose, PHFs were purified from Alzheimer’s disease brain samples using the Greenberg method (see Section 2), and PHFs were coincubated with the W-Tau peptide. After 24 h of incubation, the peptide could interact with the purified PHFs, eliminating the initial structure of the polymer. Overall, the peptide significantly disrupted or disintegrated PHFs at 24 h (Figure 4A) in a circular oligomeric manner. These oligomers coincided in diameter (Figure 4B) with those formed by the incubation of tau 2N4R with the W-Tau peptide (Figure 1E). In the presence of the W-Tau peptide, no PHFs were found (Figure 4C). Turbidity analysis of the incubation of the W-Tau peptide and PHFs revealed differences in absorbance, indicating conformational changes of PHFs into these circular oligomeric structures (Figure 4D). The incubation of PHFs for 48 or 72 h with that peptide also led to the formation of these circular structures, but not in the presence of a similar peptide (Figure S2A). Circle-like aggregates lost the ordered conformation of the PHFs, reducing their structure to tau circular oligomers.

### 3.5 W-Tau Peptide Binds to PHF Ends Facilitating Their Shapeshifting and Disintegration

**Figure 5:**
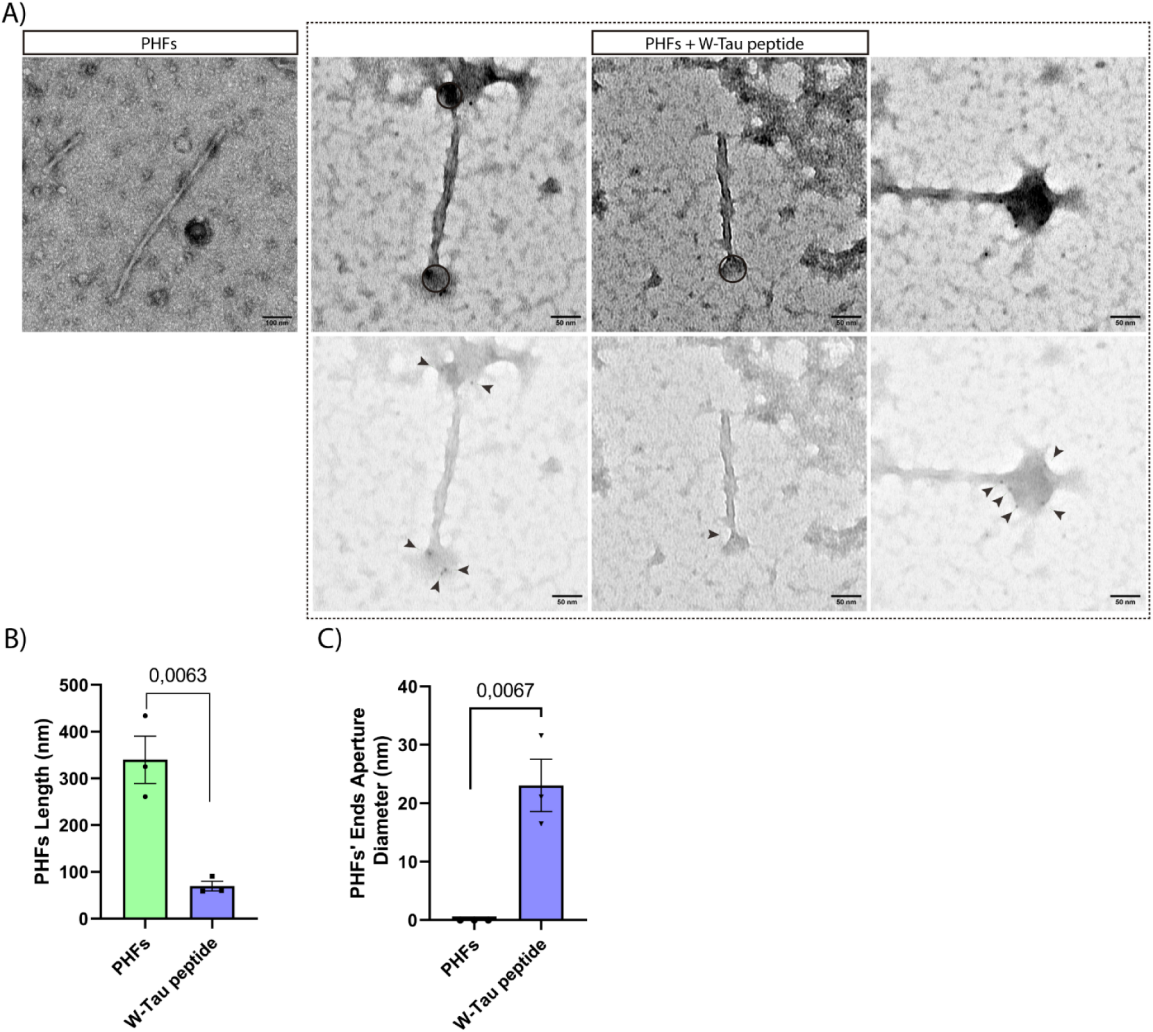
Binding of W-Tau peptide to PHFs. (A) Representative immunogold electron microscopy images of the binding of W-Tau peptide (black arrow heads point to nanogold particles that localize the peptide) to the ends of PHFs initiating degradation of PHFs. Scale bar of 100 and 50 nm. Black dots correspond to ferritin shells which are present in PHF samples.^57^ (B) Quantitative analysis of the length of PHFs. (C) Diameter measure of PHF’s end points aperture produced by W-Tau peptide. Quantitative analysis shows the mean ± SEM. *p*-vValue, represented in each graph, using two-way ANOVA followed by Student’s *t* test for comparisons. Sample size: *n* = 20 fields per replicate. Each single value in graphs represents each replicate from independent samples (technical replicates: *n* = 3).

After investigating the disaggregation of PHFs by the W-Tau peptide, the next point of interest of the study was to determine how the binding of the W-Tau peptide to PHFs occurs. The chosen method to observe these characteristics was to perform immunogold labeling, which allowed us to attach gold-labeled antibodies to the antibody that recognizes the W-Tau peptide. This enabled us to observe, through electron microscopy, the preferred positions of our peptide when binding to the PHFs. As shown in Figure 5A, high-density marks (i.e., the gold particles) are observed at the ends of the PHFs. This confirms the interaction between the PHFs and the W-Tau peptide in a specific manner, focusing on the ends of the filaments, where the disintegration of the PHFs would begin. The shapeshifting of PHFs induced by the W-Tau peptide can be observed in the fan-shaped opening at the ends and the decrease in the length of the filaments (Figure 5B), suggesting that the PHFs are beginning to disintegrate. This shapeshifting mechanism could lead to the formation of a less organized tau protein structure, resulting in a protein cluster with a circle-like conformation.

The analysis of the diameter of the initial openings of the end points of the PHFs (Figure 5C) produced by the effect of the W-Tau peptide fits as the size of the formation of the Tau oligomers previously reported.^56^ These end point openings could promote the formation of cores onto which unstructured protein derived from PHFs would aggregate.

## 4 Discussion

The novel isoform of tau, known as the W-Tau isoform, is generated by intron 12 retention of tau RNA. It has been found to play a role in the inhibition of tau aggregation and is decreased in AD.^42,43^ This intronic retention of 18 amino acids leads to the formation of the human-specific W-Tau peptide, which has been the main point of our research in this work, with a focus on its interaction with human PHFs.

In this study, we conducted *in silico* and *in vitro* investigations at both the molecular and cellular levels, which enabled us to explore the efficacy and properties of the W-Tau peptide from multiple perspectives. The *in silico* analyses, conducted using the AF3 server,^48^ provided us with an initial visualization of how the W-Tau peptide might alter interactions between tau monomers. Upon conducting *in vitro* analyses, we observed that it was indeed capable of both preventing polymerization from PHF seeds and promoting the depolymerization of PHF extracted from the brains of patients with AD. This finding suggests that the interactions predicted by computational models may not fully capture the complexity of biological systems and underscores the importance of confirming these results through more direct experimental techniques.

In the course of this study, we consistently observed the ability of the W-Tau peptide to inhibit tau polymerization. Specifically, we observed *in vitro* that the W-Tau peptide’s capacity to inhibit filament formation^47^ reduces the assembly activity of PHF seeds. The inhibition of PHF seeds’ assembly could help prevent the aggregation activity that tau exhibits in various pathologies,^58−60^ thereby reducing pathways for the accumulation of aggregates that are harmful to the physiological functioning of the CNS. Furthermore, our studies demonstrated that the peptide is capable of disassembling already formed PHFs purified from the brains of Alzheimer’s patients, providing compelling evidence of neuroprotective activity beyond mere prevention of new aggregate formation. Future studies examining the peptide’s effects on filaments from diverse tauopathies would further elucidate its broad neuroprotective potential and expand our understanding of tau pathology mechanisms across neurodegenerative disorders.

About the role of the W-Tau peptide in tau filament formation, it was described that in such polymerization process, the sequence -VQIVYK- (also known as the PHF6 motif), present at the beginning of exon 11, could promote a structural β-sheet conformation that could facilitate the formation of tau oligomers.^61−63^ When the oligomer lengthens, it could transform into granular aggregates, a possible intermediate stage in filament polymerization.^64,65^ These granular aggregates may be precursors of PHFs. Our result suggests that the interaction of the W-Tau peptide with the -VYK- motif of exon 11 prevents the formation of tau filaments but may favor the formation of granular oligomers.

The destabilization of PHFs by the W-Tau peptide from their ends results in a pattern similar to the accumulation of circular tau oligomers described by Takashima and his group.^56,65,66^ These circular tau oligomers are similar in size to the openings made at the ends of the PHFs by the W-Tau peptide. The Takashima group was the first to suggest that the formation of a circular tau oligomer precedes PHF formation, and if that is a reversible process, the W-Tau peptide will shapeshift the PHFs reversing the process of filament formation. In addition, these circular tau oligomers are found in non-AD brains, indicating a lower impact on neuronal pathology compared with PHFs, as previously hypothesized.^56,65,66^ This opening at the ends, considering the decreased analyzed distances of the filaments, could explain the disruption of PHFs in the presence of the W-Tau peptide.

**Figure 6:**
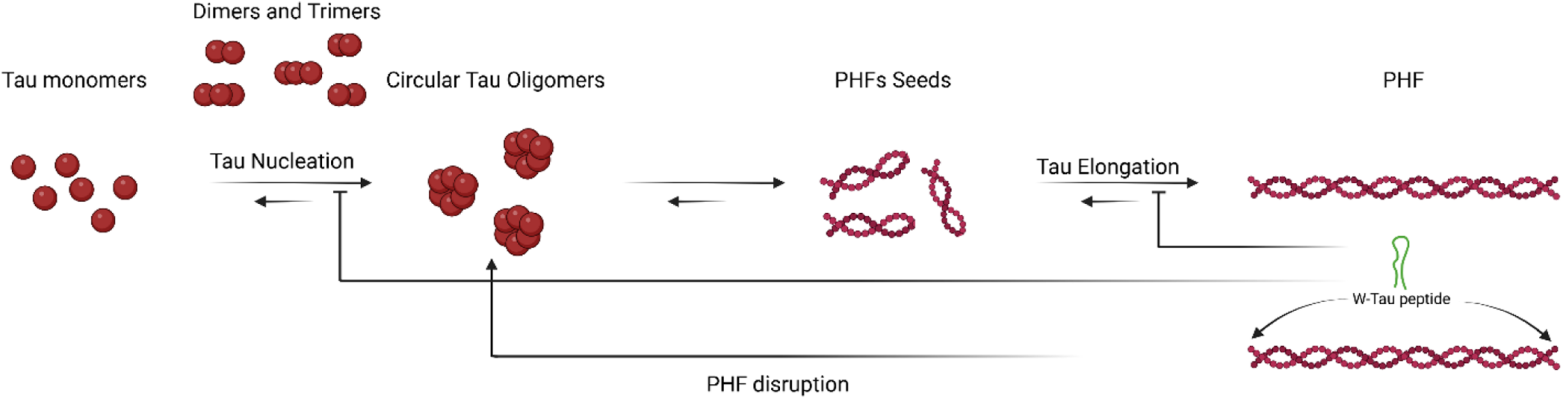
Graphical abstract of the inhibition of Tau filaments’ assembly and PHFs’ disruption by the W-Tau peptide. The pathological aggregation of tau begins with a process of nucleation of the monomers into oligomeric structures, a process that is reversible but highly favored toward oligomer formation. These oligomeric structures elongate and form filamentous structures called PHFs. This aggregation process is modified by the W-Tau peptide, which is able to inhibit both nucleation and elongation of the protein, as well as disrupting the already existing PHFs, leading to oligomeric tau structure formation.

In summary, our findings highlight the significance of the W-Tau isoform and the W-Tau peptide (Figure 6), their natural presence, and neuroprotective capacity. On the other hand, the W-Tau isoform is present in a lower amount compared to the well-known six CNS tau isoforms. However, the W-Tau isoform has the potential for enhanced expression, and if this is the case, it will have a therapeutic effect.^44^ Further exploration of its mechanism of action *in vivo* and its potential application in disease models remain crucial areas of research for advancing the field of neurology.

## 5 Conclusion

In this study, we demonstrated that the W-Tau peptide not only inhibits Tau aggregation but also depolymerizes preexisting paired helical filaments extracted from the brain samples of Alzheimer’s disease patients. As a result, the W-Tau peptide holds potential as a candidate for future therapeutic strategies in the treatment of tauopathies.

## Data Availability

The data sets generated during the current study are available in the *figshare* repository, https://figshare.com/articles/dataset/RawData_Shapeshifter_W-tau_peptide_inhibits_tau_aggregation_and_disintegrate_paired_helical_filaments/27249207?file=49849401.

## Supporting Information Available

W-Tau vs Control peptide comparison (Figure S1); EM imaging and quantification (Figure S2) (PDF)
