# Supplementary material for: Shapeshifter W-Tau Peptide Inhibits Tau Aggregation and Disintegrates Paired Helical Filaments
Source: Biochemistry. 2025 Mar 27;64(8):1841–51. doi: 10.1021/acs.biochem.4c00809 (PMC12004447; doi:10.1021/acs.biochem.4c00809)
Supplement: Supplementary file 1 — bi4c00809_si_001.pdf [file bi4c00809_si_001.pdf]

Supporting Information

Shapeshifter W-Tau peptide inhibits tau aggregation and disintegrates paired helical filaments

Indalo Domene-Serrano[1,2], Raquel Cuadros[1], Vega García-Escudero[1,4], Francisco Vallejo-Bedia[1], Ismael Santa-María[2], Laura Vallés-Saiz[1], Félix Hernandez[1] & Jesús Avila[1,3]*

[1]Centro de Biología Molecular Severo Ochoa, CSIC-UAM, 28049 Madrid, Spain

[2] Facultad de Ciencias Experimentales, Universidad Francisco de Vitoria, Pozuelo de Alarcon, 28223 Madrid, Spain.

[3]Center for Networked Biomedical Research on Neurodegenerative Diseases (CIBERNED), Instituto de Salud Carlos III, 28029 Madrid, Spain

[4]Departamento de Anatomía, Histología y Neurociencia, School of Medicine, Autonoma de Madrid University (UAM), Arzobispo Morcillo, 4, 28029 Madrid, Spain.

*Corresponding author: Jesús Avila, Centro de Biología Molecular Severo Ochoa, CSIC-UAM, C/Nicolás Cabrera, 1. 28049 Madrid, Spain. E-mail: javila@cbm.csic.es Phone: +34 911964564 ORCID: https://orcid.org/0000-0002-6288-0571

Supplementary Figure 1

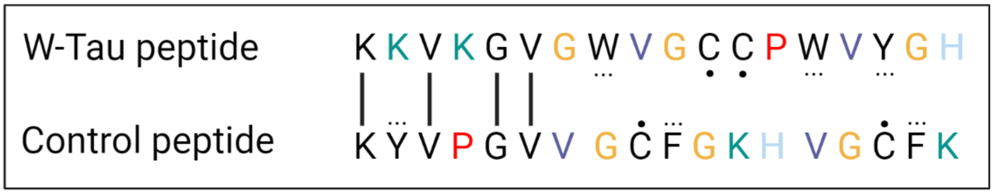

Figure S1 A) Differences between the W-Tau peptide and the Control peptide. Each peptide has 18 amino acids, they coincide in the position of four amino acids (straight bar). Both peptides contain two cysteines (single dots), structurally and functionally important. Each has the same number of non-acidic amino acids (consecutive dots): W, W, Y in the W-Tau peptide and Y, F, F in the control peptide. Additionally, the remaining amino acids are shared between the two peptides but in different positions (varied colors). These sequence similarities, along with the complete differences observed experimentally between the two peptides, demonstrate that the effects exerted by the W-Tau peptide on the tau protein and its aggregates are entirely specific.

Supplementary Figure 2

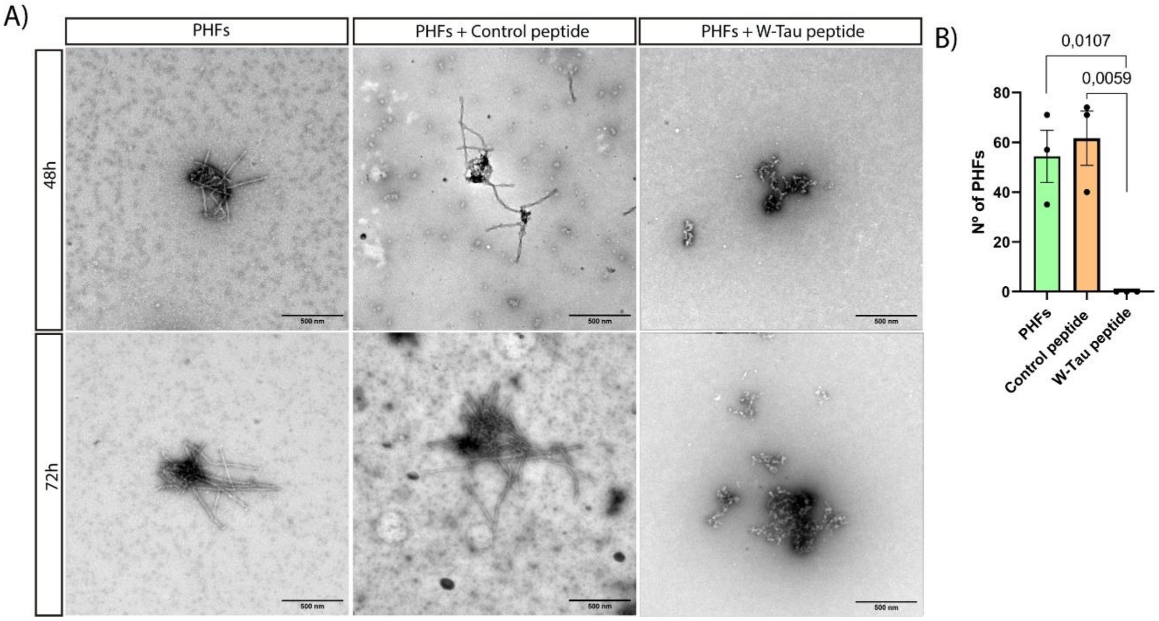

Figure S2 A) Electron Microscopy representative images of PHFs (Control) and the action of W-Tau peptide and Control peptide over PHFs at 48 and 72 hours. Scale bar of 500 nm. **B)** Quantification of number of PHFs founded per replicate at 72 hours. Number of paired helical filaments found per field. Each single value in graphs represent each technical replicate. Quantitative analyses show the

mean ± SEM. * $p < 0.05$; ** $p < 0.01$; *** $p < 0.001$ using two-way ANOVA followed by Student's t-test for comparations. Sample size: n= 20 fields per replicate. Each single value in graphs represent each technical replicate (n=3).
